# On Midwives and Accoucheurs

**Published:** 1816-02

**Authors:** Joseph Adams


*For the London Medical and Physical Journal.*

### *On Midwives and Accoucheurs*; by JOSEPH ADAMS, M.D.

I HAVE been much interested in the controversy of some of your learned correspondents concerning the obstetric art, and shall feel obliged by your admitting a few loose

 thoughts thoughts on the subject, if not inconsistent with the plan of your Journal.

I would first remark, not only that *accoucheurs* are of recent origin, but that no language with which I am acquainted has a primitive word for a male practitioner in that art. The Hebrew midwives were all female, as far as we may judge by the sacred text. In Greek, we find Hippocrates feminizing an old fashioned word applied to a healer, and even to a botcher; and other writers using a term as often applied to the nurse. The noun *obstetrix*, in Latin, has, I believe, no masculine. In modern languages, the Germans have two expressions, both of which seem to refer to labour-pains or groanings, and both are feminine. The Italians have dropped the old Latin term, and use *commere*, which is indifferently applied to a nurse or gossip attending at those periods. The Portuguese and Spaniards, who, next to the Italians, retain most of the original Latin, have *commadre*, used in the same senses, and also as a god-mother. They have, indeed, the term medico, or chirurgeon-parteiro, but these are evidently of modern invention, from *partire*, or the Latin *partum* supine of *pareo.*

The proper French term is *sage femme*, so called, not only on account of her supposed knowledge, but because she is empowered, in cases of necessity, to baptize. For this purpose, she does not always wait for the birth of the child; but if the labour is lingering, and on that account likely to prove fatal to the infant, it is, in many parts of the continent, baptized by dipping the midwives finger into water, which is brought into contact with any part of the child: the woman then pronounces *In nomine patris*, &c. concluding by proclaiming "The child is baptized." Of late years the French have adopted the word *accoucheur*, or *chirurgien accoucheur*, to signify the office when conducted by men.

I am not sufficiently acquainted with the Anglo-Saxon to ascertain whether *gossip* was among them the only term for those who exercised that office, and afterwards midwife added, to distinguish that lady from the other attendants. The latter term, however, seems plainly to show to which sex it was confined; and, if I am right, can there be a greater absurdity than to talk of a *man-midwife?* But our term *lying-in*, or, according to modern delicacy, *confinement*, is ill suited to form into a substantive: hence we have been under the necessity of using the French word *accoucheur.*

From what has been said, I conceive, it will not be questioned that the introduction of accoucheurs is of modern date, probably first begun by the French, who, it must be be admitted, were the masters of all Europe in the early practical improvement of surgery. It is, however, certain that physicians and (as soon as surgery was reduced to an art) surgeons attended to all the circumstances of gestation and parturition, and that in many difficult cases they were consulted. The only remaining inquiry, therefore, is, whether the modern practice of applying in all cases to men is an improvement? for to say that they are always necessary would be an absurdity.

In pursuing such a question, our first business is to ascertain the duties of the office. These are, an accurate knowledge of the parts, of the progress of gestation and parturition, of the diseases arising from the first, and the interruption to the last. These, from whatever causes, would naturally lead to the proper mode of relief, with or without instruments. This comprehends likewise all the incidents by which we may distinguish genuine from preternatural gestation, and also the various and very complicated diseases attending each.

If women are not as well informed on these subjects as men, it is, probably, only because they are not so well instructed. Interdicted from using instruments, and always taught to call the assistance of the other sex under every difficulty, their chief attention is to the natural progress of the labour, and the signs by which they may judge of any impediment. I will, therefore, for argument sake, suppose them in all respects as well instructed as the men, and then inquire whether such instructions are all that is necessary to constitute a complete accoucheur?

Surely every reader must be aware of two grand qualifications which, unless we could alter the race, must for ever remain deficient in the female. I mean courage and corporeal strength. That there are females who possess both, cannot be questioned; but the proportional number is few, and those few are not always such as we should fix on to attend their own sex during the most interesting period of their existence. But without courage, how can we expect the early application of those means which, on some occasions, can only be delayed at the certain loss of two lives. Corporeal strength is more necessary in this than in any other branch of our laborious profession. Labours more commonly occur at night than during the day; and, if they do not occur at the former period, still the gloom and apprehension of the patient and her attendants are always increased, so as to render nocturnal calls very frequent. A woman cannot always answer such calls without some protection, tection, which adds much to the difficulty and expence of her attendance; and it is only during a comparatively short period of her life that she can be equal to the fatigues attending her vocations. Another very serious inconvenience has arisen from this, which I am constrained to mention without any general imputation on the sex. After or under considerable fatigue, she will always find some of her sex at hand to offer the ready but dangerous relief of the dram-bottle. Whatever may be her inclinations, not to say resolutions, it will be scarcely possible to resist at all times, and it is unnecessary to add how gradually this habit steals on both sexes with advancing years. How much more must it be, then, where there is the addition of continual solicitation, and often a sufficient apology for complying. That such was the case with midwives, and one of the causes of their discontinuance, is notorious. Nor can it be entirely imputed to the effects of a northern climate, and the facility with which ardent spirits are procured, since the invention of distillation from malt. In Rome, and probably in Athens, long before even the distillation of brandy was known, midwives were accused of this habit. One familiar instance occurs to me in Terrence. The scene of his *Andria* is well known to be in Athens; but, supposing that he has introduced Roman customs, the question will not be materially altered. In this play an infant is born, previously to which we have the description of the *obstetrix* and nurse. In the fourth scene of the first act, the servant addresses the nurse, telling her that she heard her orders to procure Lesbia the midwife. She then adds that " Lesbia is such a tippler, and so inconsiderate, as not to be trusted in a first labour. However," says she, " I'll fetch her." As she is going she adds aside to the audience—" Importunitatem spectate aniculæ, quia *compotrix* ejus est—see how mal-apropos the old creature is, merely because they are pot-companions."

Thus it appears to me that for the most part there seems no remedy against the additional fatigue which this branch of the profession has brought on the country practitioners. At the same time, I conceive, it would be always to their advantage to select, if they can find such, the most judicious among those women who are in the habit of assisting their poor neighbours, and give them such general instructions, in the form of aphorisms, as may enable them to distinguish preternatural presentations at an early period. By these means, the surgeon might frequently be relieved for several hours, and even allowed to sleep till morning, without the loss of his fee in such families as are worth attending. Even the good good management of such a female would reflect credit on her instructor; and the general progress of gossip being often directed by these women, would run very much in favour of the man who treated them like reasonable beings.

